# From gradients to cognition: linking cortical manifolds to brain flexibility and disorder

**DOI:** 10.3389/fcogn.2025.1690469

**Published:** 2025-12-10

**Authors:** Joseph Y. Nashed, Ryan Sandarage, Christopher R. Pasarikovski, Jason P. Gallivan, Douglas J. Cook

**Affiliations:** 1 Centre for Neuroscience Studies, Queen's University, Kingston, ON, Canada; 2 Faculty of Medicine, Department of Surgery, Division of Neurosurgery, University of Ottawa, Ottawa, ON, Canada; 3 Department of Neurosurgery, Queen's University, Kingston, ON, Canada; 4 Department of Psychology, Queen's University, Kingston, ON, Canada; 5 Department of Biomedical and Molecular Sciences, Queen's University, Kingston, ON, Canada

**Keywords:** manifold, gradients, neuroimaging, cognition, learning, stroke

## Abstract

Traditional neuroscience describes the cerebral cortex as a mosaic of discrete, functionally specialized regions. However, a complementary view has emerged, demonstrating that the brain is also organized along continuous gradients that capture large-scale transitions in connectivity, microstructure, and function. These gradients, derived using dimensionality reduction techniques on neuroimaging data, provide a low-dimensional manifold framework that unifies our understanding of how cortical architecture supports cognitive flexibility, learning, and clinical disorders. In this review, we integrate evidence from genetics, phylogeny, development, and multimodal neuroimaging to outline how macroscale gradients emerge from underlying biological constraints, become progressively decoupled from local microstructure in transmodal cortex, and dynamically reorganize during cognitive and clinical states. We further discuss how this framework provides new insights into individual differences, disease mechanisms, and recovery following brain injury. By bridging anatomy, function, and behavior, gradient-based approaches offer a powerful lens for mapping the architecture of human cognition and its disruption in disease.

## Introduction: cortical gradients as a framework for cognition

Traditional neuroscience has long conceptualized the cortex as a number of discrete, functionally specialized regions (Brodmann, 1909; Felleman and Van Essen, 1991; Mesulam, 1998). This framework, supported by lesion studies, task-based neuroimaging, and cytoarchitectonic mapping, has successfully linked specific brain regions to well defined sensory, motor, and cognitive functions (see Poldrack, 2007; Rorden and Karnath, 2004). This parcellation based view, while foundational, offers a limited perspective on how distributed networks give rise to complex behavior, a gap that has been addressed by recent advances in network science and dimensionality reduction techniques applied to large-scale datasets.

**Figure 1:**
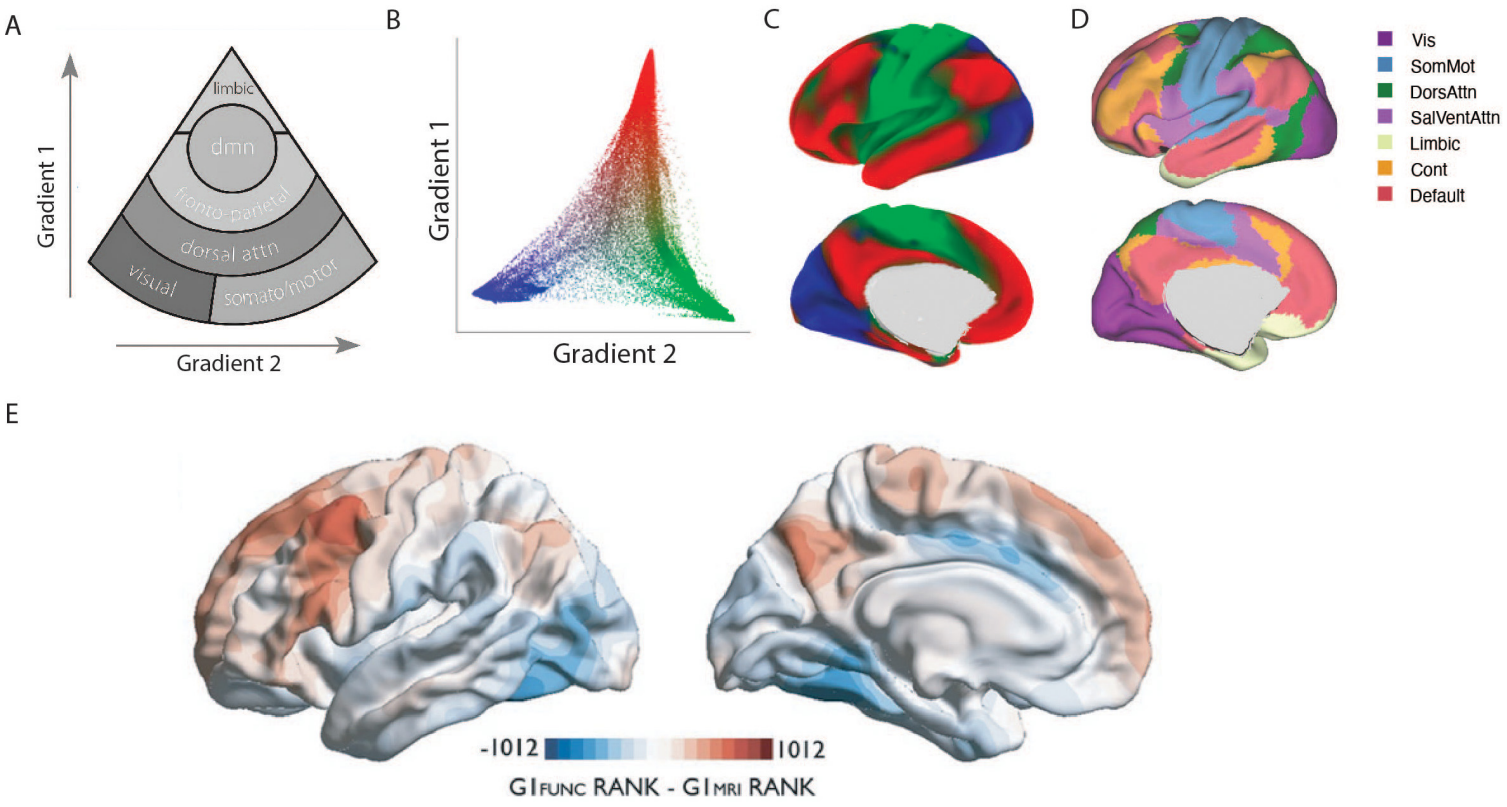
Hierarchical organization of cortical gradients and underlying microstructure. **(A)** This schematic illustrates the two principal cortical gradients derived from multimodal neuroimaging. Gradient 1 (vertical axis) extends from unimodal sensory and motor regions at the base to transmodal association cortices, with the Default Mode Network (DMN) at the apex, reflecting a shift from externally oriented processing to abstract, internally directed cognition. Gradient 2 (horizontal axis) differentiates networks along a secondary axis, separating somatomotor and visual networks. Modified from (Margulies et al., 2016). **(B)** A scatter plot of the first two connectivity embedding gradients. Gradient 1 extends between primary sensorimotor and transmodal regions (red). Gradient 2 separates somatomotor and auditory cortex (green) from visual cortex (blue). Histograms depicting the distribution of values are presented on the respective axes. **(C)** Colors from the scatter plot are presented on the cortical surface for anatomical orientation. **(D)** Illustration of 7-network Yeo parcellation for comparison of cortical gradients (Adapted from (Yeo et al., 2011)). **(E)** Divergence between microstructural and functional gradients in association cortices. Comparison of node ranks along the principal microstructural gradient (blue) and the principal functional gradient (red) reveals their close alignment in primary sensory regions but increasing divergence in transmodal cortices. Note that GI reflects the Gradient Index which reflects the “upward” or “downward” shifts in relative positions of functional vs. microstructural gradient space, indicating the degree of structure–function dissociation. Prefrontal and precuneus regions shift upward in functional gradient space, while posterior inferior temporal and midcingulate regions shift downward, highlighting that higher-order association networks are less constrained by local microarchitecture and occupy distinct positions in the functional hierarchy (Adapted from Paquola et al., 2019).

In recent years, a complementary framework has emerged, proposing that the cortex is organized along continuous gradients rather than sharp boundaries. Typically derived by applying non-linear dimensionality reduction techniques (e.g., diffusion map embedding) to functional connectivity matrices, these gradients describe smooth transitions in connectivity, microstructure, and function across the cortical surface, offering a low-dimensional scaffold that unites diverse aspects of brain organization (Huntenburg et al., 2018; Margulies et al., 2016; Vos De Wael et al., 2020). The principal gradient, for example, extends from unimodal sensory and motor regions to transmodal association cortex, with the Limbic and Default Mode Networks (DMN) at its apex (See Figure 1). This axis captures a fundamental shift from externally oriented, stimulus-driven processing to internally directed, abstract cognition. By embedding the cortex in a continuous gradient space, researchers can link large-scale anatomy to functional hierarchies. Several measurable properties can be derived from these embeddings, including eccentricity (distance from the manifold center, indexing a region's segregation), node rank (its hierarchical position along a gradient), and displacement (reconfiguration across conditions) See Box1. This framework also provides a powerful lens for examining how the brain flexibly supports cognition and how these organizational principles are altered in clinical disorders.

Box 1 **Key terms and computation**

**Manifold:** A low-dimensional representation of multivariate brain organization derived from similarity matrices (e.g., functional connectivity, cortical wiring, microstructure).

**Gradient:** A principal axis within the manifold capturing smooth transitions in cortical organization.

**Eccentricity:** A scalar measure reflecting a region's distance from the manifold centroid—quantifying its degree of segregation/integration or transmodal specialization.

**Excursion:** The displacement of a region's embedding between conditions or states, reflecting functional reconfiguration.

**Node Rank:** A region's relative position or order along a given gradient axis, reflecting its placement within the brain's hierarchical or functional continuum.

*Note: Each metric is computed from the group-level manifold derived using diffusion map embedding or PCA of functional connectivity matrices*.

## The anatomical and genetic scaffold of cortical gradients

The large-scale organization of the cortex is not arbitrary but is anchored in deep biological constraints shaped by genetics, evolution, and development. Valk et al. (2020) demonstrated that cortical thickness is organized along posterior–anterior and inferior–superior axes, patterns that are conserved across species and strongly influenced by genetics (Valk et al., 2020). These gradients align with the dual-origin theory of cortical evolution, suggesting that human cortical organization reflects ancestral and derived phylogenetic axes.

Phylogenetic analyses indicate that gradients of cortical thickness parallel gradients of laminar complexity and cytoarchitecture, reflecting coordinated evolutionary and developmental processes. Across primate species, regions that have undergone the greatest evolutionary expansion, particularly multimodal association areas, display increased supragranular differentiation, greater laminar heterogeneity, and a relative enrichment of intracortical and long-range connections (Rakic, 2009). These microstructural and phylogenetic patterns align with gradient-based hierarchies that distinguish primary sensory and motor cortices from higher-order transmodal regions (Buckner and Krienen, 2013). Together, these convergent lines of evidence suggest that phylogenetic expansion, laminar specialization, and cytoarchitectural differentiation act in concert to shape the macroscale gradients that underpin flexible cognition and integrative cortical function.

The anatomical and genetic scaffold of cortical gradients also unfold across the lifespan. (Sydnor et al. 2021) extended this framework developmentally, demonstrating that association cortices mature along a multimodal sensorimotor–association axis defined by coordinated gradients in cortical thickness, T1w/T2w-derived myelination, and externopyramidization. These gradients capture a progression from early-developing, heavily myelinated sensory areas to later-developing, less-differentiated association cortex, reflecting increasing laminar complexity and long-range integrative capacity that underlies functional specialization and flexibility in transmodal regions (Sydnor et al., 2021). This developmental reorganization helps establish the macroscale scaffolding that supports functional specialization and flexibility in transmodal cortex. Beyond development, recent work has connected these macroscale patterns to their underlying microstructural foundations. Meng et al. (2022) identified a functional similarity gradient in human cortex that aligns closely with cytoarchitectonic and laminar differentiation principles (Meng et al., 2022). By integrating functional connectivity with histological features, their study demonstrated that regions positioned at the apex of the gradient, such as transmodal association cortex, exhibit greater laminar complexity and more diverse connectivity profiles, while primary sensory regions remain tightly coupled to simpler microarchitectural motifs. This convergence of evidence suggests that macroscale cortical gradients are not a superficial functional phenomenon but arise from the coordinated influence of deep biological constraints, including cellular architecture, evolutionary expansion, and genetic factors. This multi-scale interplay provides a foundational bridge linking molecular, structural, and functional levels of brain organization.

This developmental trajectory corresponds with increasing eccentricity in manifold space, consistent with evidence that later-developing, less-myelinated association areas occupy more transmodal positions within the cortical hierarchy (Sydnor et al., 2021; Paquola et al., 2019). During adolescence, as long-range connectivity and laminar differentiation continue to mature (Park et al., 2021), these association cortices shift outward along the principal functional gradient, reflecting growing segregation from unimodal networks and greater integrative capacity. This developmental expansion of manifold eccentricity parallels the protracted myelination and synaptic pruning observed in frontoparietal and default mode systems (Valk et al., 2020), which are thought to underlie the emergence of cognitive flexibility and abstract reasoning in adulthood. Together, these findings suggest that structural maturation and functional embedding co-evolve to shape the hierarchical architecture of the cortex.”

## Functional and microstructural dissociation in transmodal cortex

The relationship between cortical structure and function varies systematically across the brain, becoming increasingly complex in higher-order association areas. This variability is most apparent in the phenomenon of structural–functional dissociation. Paquola et al. (2019) demonstrated that while microstructural gradients, such as myelin density and laminar differentiation, follow a clear sensory–fugal hierarchy, functional connectivity gradients increasingly decouple from this scaffold in transmodal regions, including the Default Mode and Frontoparietal Networks (Paquola et al., 2019). Meta-analytic decoding linked these dissociated regions to higher-order functions such as social cognition and cognitive control, suggesting that flexible network engagement depends on reduced structural constraints.

To better quantify this dissociation, subsequent work has focused on integrating multiple scales of brain organization. Paquola et al. (2020) introduced a multi-scale cortical wiring space that combines histological and *in vivo* MRI data, revealing that transmodal regions lie at the extremes of this wiring space (Paquola et al., 2020). These extremes reflect greater geodesic distances in cortical wiring, indicating that transmodal regions are less tethered to local microstructure and can flexibly integrate distant information, a hallmark of structural–functional dissociation.

This finding reinforces the view that structural–functional dissociation is a hallmark of association cortex organization and provides a rigorous framework for measuring it. Importantly, this dissociation is not static, it evolves over development. Park et al. (2021) showed that adolescence is characterized by manifold expansion in transmodal networks, reflecting structural reorganization that contributes to increased structure–function decoupling and cognitive flexibility (Park et al., 2021). A recent review by Bernhardt et al. (2022) synthesized this evidence, arguing that transmodal cortex achieves its adaptive capacity through this progressive loosening of structural tethering (Bernhardt et al., 2022). This body of evidence converges on a common principle: structural–functional dissociation is a defining property of association cortex, allowing transmodal regions to flexibly integrate information across multiple functional domains and support complex cognition.

## Gradients and cognitive flexibility

Cortical gradients and their manifold representations provide an essential framework for understanding higher-order cognition. Rather than being confined to localized circuits, cognition emerges from the interaction of large-scale networks organized along low-dimensional axes. The principal gradient's axis is not merely a descriptive feature but is thought to reflect a fundamental principle of neural computation: hierarchical abstraction. At the unimodal end, neural representations are tightly coupled to specific sensory modalities, processing concrete, immediate features of the external world. As one moves along the gradient toward the transmodal apex, information becomes progressively integrated across modalities and abstracted from immediate sensory input. This allows transmodal regions, such as the default mode network, to operate on abstract, memory-based, and internally generated information, a process essential for functions like planning, reasoning, and mental simulation. Transmodal cortices, positioned at the apex of the principal gradient, are ideally suited for this role, supporting mental simulation, abstract reasoning, future planning, and social cognition (Huntenburg et al., 2018; Margulies et al., 2016).

**Figure 2:**
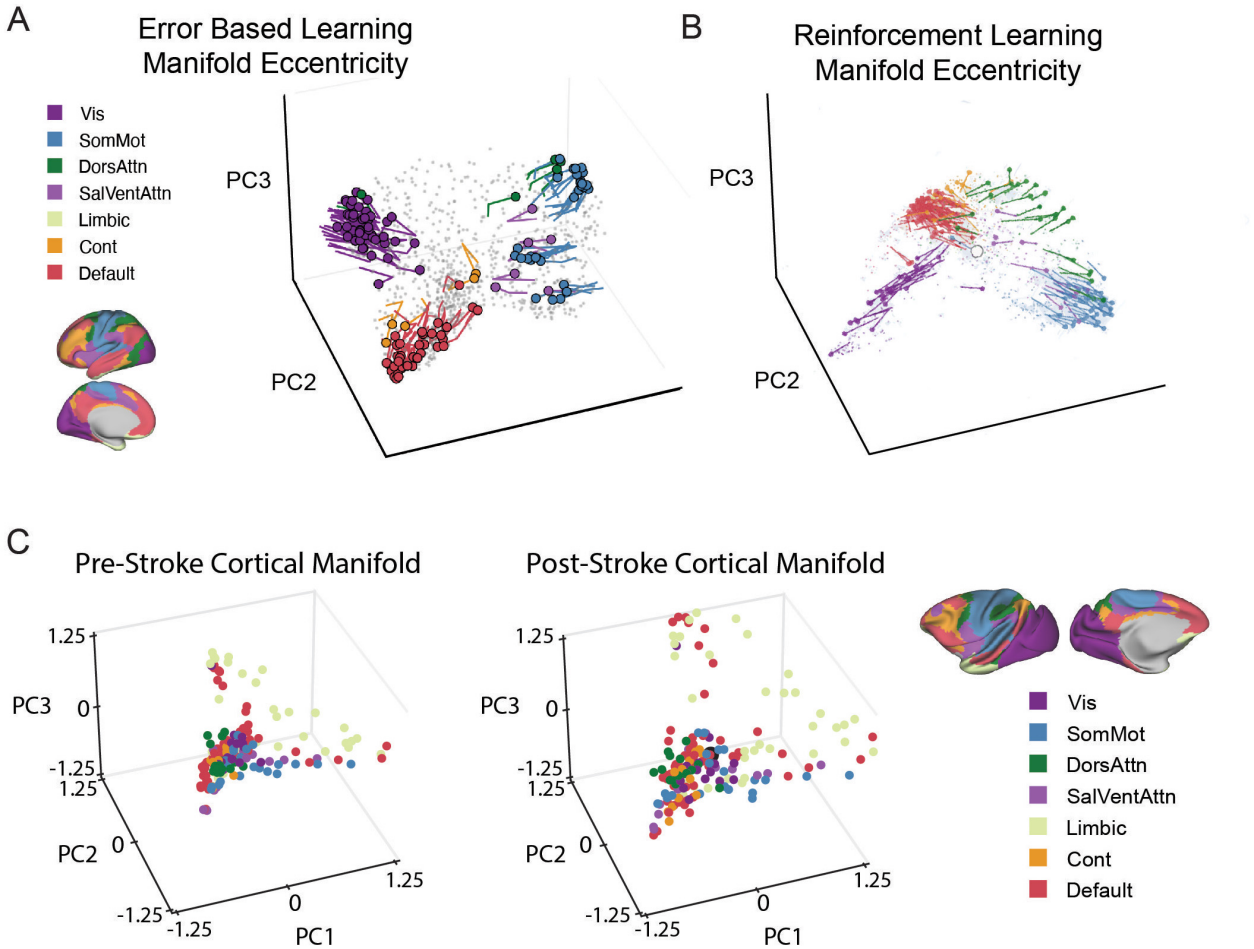
Changes in Manifold eccentricity in learning and pathology. **(A)** Temporal trajectories of regions implicated in adaptation learning from early to late learning. Adapted from Gale et al. (2022). **(B)** Another example of changes in manifold eccentricity from Nick et al. (2024). Temporal trajectories of key regions in low-dimensional space expand away from the centroid from baseline to early learning in a reward-based learning task. Adapted from Nick et al. (2024). For both A and B Colored circles indicate each region's initial position during Baseline, and the traces show the unfolding displacement temporally through learning. Each region is colored according to its functional network assignment. In other words, the plotted lines represent trajectories of regions demonstrating significant displacement from the centroid across task epochs, based on statistical analysis. **(C)** Pathological changes in manifold structure: In a macaque model of ischemic stroke (Nashed et al., 2024). Illustrated that cortical manifolds in low-dimensional space in post-stroke (right) expand considerably compared to pre-stroke (left). The figure illustrates the same functional network denoted in the yeo 7-network atlas denoted above in **(A)** and **(B)**, except mapped onto the macaque brain. Each data point represents a single brain region, with its location in the three-dimensional manifold space being determined based on its loading onto each of the PCs. In all panels, functional connectivity (FC) matrices were computed from resting-state (Panel **C**) or Task-specific windowed sections of fMRI time series (Panels **A** and **B**), using region-wise BOLD signal covariance. The resulting FC matrices were row-wise thresholded and converted into affinity matrices via cosine similarity. Low-dimensional manifold embeddings were derived using principal component analysis (PCA), and aligned to a reference (template) manifold using Procrustes alignment to permit cross-subject and cross-epoch comparisons. Eccentricity was computed as the Euclidean distance of each region from the manifold centroid (i.e., [0, 0, 0]), reflecting its degree of functional segregation versus integration. Displacement represents the change in a region's position in this manifold space across conditions (e.g., baseline vs. learning, or pre- vs. post-stroke). In other words, manifold expansions reflects increasing network segregation, while contractions reflects greater network integration.

Evidence from learning studies supports this view. Areshenkoff et al. (2022) demonstrated that excursions from intrinsic manifold structure during learning involve not only motor-related regions but also higher-order association networks (Areshenkoff et al., 2022). Building on this, Gale et al. (2022) showed that cortical manifolds exhibit distinct phases of expansion and contraction that correspond to specific stages of learning (See Figure 2). Early manifold expansions, which reflects increasing network segregation, are linked to error-driven adaptation and broad recruitment of both sensorimotor regions and higher-order association cortices, including frontoparietal and default mode areas, reflecting the engagement of cognitive strategies. Later contractions, which reflects greater network integration, suggest the refinement and stabilization of these networks as task execution becomes more automatic and less reliant on explicit control. These dynamics highlight the flexible reorganization of cortical systems to meet changing task demands (Gale et al., 2022).

Motivational factors also shape these manifold dynamics. Nick et al. (2024) demonstrated that reward-based learning drives widespread reconfigurations of cortical manifolds that include frontoparietal and default mode networks (See Figure 2). These changes suggest that motivational signals actively shape manifold structure, enabling the integration of cognitive control, value processing, and goal maintenance during learning. This evidence places gradient-based organization at the core of how the brain dynamically links motivation and control (Nick et al., 2024).

Finally, subcortical–cortical interactions add another layer to this framework. Zhu et al. (2025) showed that cerebellar–cortical manifold contractions play a critical role in motor reinforcement learning, underscoring that subcortical-cortical interactions contribute to both motor and cognitive learning processes. Given the cerebellum's known involvement in prediction, error monitoring, and higher-order cognition, these findings suggest that cerebellar-driven manifold changes may extend beyond motor control, influencing adaptive cognitive processes such as planning and strategic adjustment (Zhu et al., 2025).

Collectively, these findings position gradients and manifolds as a bridge between the brain's intrinsic organization and the flexible behaviors required for learning and adaptive cognition. This perspective naturally extends to clinical contexts, where disruptions in gradient organization have been shown to underlie both psychiatric conditions and network-level reorganization after brain injury, as described in the following section.

## Disruption of gradient architecture in clinical populations and stroke

Alterations in cortical gradients are observed across neuropsychiatric disorders and after focal brain injury, suggesting that disruptions in low-dimensional brain organization contribute to both clinical symptoms and recovery mechanisms. In autism spectrum disorder, Hong et al. (2019) reported reduced differentiation between sensory and transmodal regions, with flattened gradients and disrupted stepwise connectivity associated with deficits in sensory integration and social cognition (Hong et al., 2019). Similarly, Xia et al. (2022) demonstrated that major depressive disorder is characterized by compression of the principal gradient, reducing functional separation between unimodal and transmodal areas (Xia et al., 2022). This gradient contraction was linked to gene expression profiles related to synaptic signaling and predicted treatment response. Similar alterations have been observed in schizophrenia, where a disruption of the principal gradient is thought to reflect a blurred distinction between internal thought and external sensory processing, contributing to core symptoms like psychosis (Xiang et al., 2025).

Stroke provides an additional natural model for studying the effects of perturbations on gradient architecture. Bayrak et al. (2019) showed that post-stroke functional reorganization aligns with gradient-defined distances rather than anatomical proximity, highlighting the biological relevance of gradients (Bayrak et al., 2019). Complementing this, Nashed et al. (2024) demonstrated in a macaque stroke model that changes in DMN and limbic embeddings, rather than motor cortex alone, predict behavioral recovery (Nashed et al., 2024) (See Figure 2). These findings indicate that higher-order transmodal regions and their integration along gradient axes play a crucial role in network-level plasticity and functional restoration. These studies emphasize that disrupted gradients reflect a shared reorganization of macroscale brain architecture that constrains cognition but also offers pathways for recovery.

While gradient-based frameworks offer exciting potential as biomarkers for neuropsychiatric conditions and recovery trajectories, their clinical utility remains preliminary. Current studies often demonstrate group-level differences in gradient architecture across diagnostic categories or in response to interventions. However, the predictive performance of these metrics at the individual level is not yet robust. Key challenges include limited test–retest reliability of functional gradients, especially in association cortex (Knodt et al., 2023; Gao et al., 2022), vulnerability to head motion and physiological artifacts, and inconsistent generalization across imaging sites or acquisition protocols (Xifra-Porxas et al., 2021; Satterthwaite et al., 2019; Murphy et al., 2013). Furthermore, gradient-derived metrics are sensitive to methodological variation, including preprocessing steps, parcellation schemes, and dimensionality reduction choices, which can impact reproducibility (Nenning et al., 2024; Kong et al., 2023; Hong et al., 2020). As such, while these findings highlight intriguing avenues for future biomarker development, caution is warranted in overinterpreting gradient metrics as ready for clinical translation. Systematic benchmarking of reliability, generalizability, and noise sensitivity will be essential for advancing their diagnostic and prognostic value.

## Caveats in gradient derivation and interpretability

While many of the cited studies leverage common dimensionality reduction techniques to derive cortical gradients or manifold embeddings, it is important to acknowledge that the underlying inputs are often heterogeneous (Wang et al., 2025; Paquola et al., 2019; Bernhardt et al., 2022). For example, gradients may be computed from resting-state or task-based functional connectivity, cortical microstructure measures, or cortical wiring models that integrate multiple data types (Wang et al., 2025; Paquola et al., 2019; Bernhardt et al., 2022). Each of these inputs, along with choices in kernel construction, embedding algorithms, and preprocessing pipelines, can result in distinct rotations, scalings, or even reconfigurations of the gradient axes (Kong et al., 2023; Kim et al., 2024). Consequently, while broad organizational patterns may converge across methods, the direct interpretability and alignment of specific gradients should not be assumed. Future work comparing gradients across modalities within subjects, or normalizing embedding spaces, is necessary to validate consistency. Interpretations should be made within the context of each gradient's construction, and caution should be taken not to treat low-dimensional embeddings from diverse inputs as functionally or anatomically interchangeable.

Finally, while gradients provide a useful low-dimensional abstraction of brain organization, recent critiques highlight limitations in this framework. In particular, Petersen et al. (2024) argues that cortical architecture is better understood as a set of discrete, developmentally determined areas. Their analysis suggests that gradient models may obscure the modular, areal basis of brain structure, especially when interpreted as continuous anatomical substrates. Gradients, in this view, are statistical summaries that should not be conflated with direct representations of cortical function or structure. Future work should integrate these critiques by combining areal models with gradient-based approaches, leveraging each to better map the complexity of brain organization. Researchers should also exercise caution when generalizing gradient findings across data modalities or assuming their biological specificity.

## Conclusion and future directions

Cortical gradients provide a powerful framework for unifying structural, functional, genetic, and developmental dimensions of brain organization. By moving beyond discrete parcellations, gradient-based approaches offer a low-dimensional map that links anatomical constraints with cognitive flexibility and highlights how these relationships are disrupted in disease.

This perspective underscores how evolutionary expansion, genetic influences, and developmental maturation shape macroscale gradients, which in turn scaffold transmodal regions central to adaptive thought and behavior. Functional decoupling from microstructural constraints in these regions supports flexible integration across networks, while alterations in gradient architecture characterize diverse clinical conditions, from autism to major depression, and predict patterns of recovery after stroke. Although prior work has highlighted the roles of development and genetics in shaping cortical organization, much of the research cited in this review extends this perspective by linking these influences to measurable gradient properties, such as manifold eccentricity and displacement, which provide a compact readout of a region's position in the brain's functional hierarchy. In particular, regions with higher eccentricity tend to be those that are later-developing, less myelinated, and more transcriptionally distinct, consistent with prior gradient-informed developmental frameworks (e.g., Paquola et al., 2019; Park et al., 2021).

One final consideration concerns how manifold approaches relate to other established frameworks for studying brain organization, such as graph theory. Whereas graph-theoretic analyses describe network topology in terms of discrete nodes, edges, and modular communities, manifold frameworks instead model the cortex as a continuous geometric landscape. This representation captures gradual transitions in connectivity and function, allowing hierarchical and representational relationships to be quantified in low-dimensional space. In doing so, manifold analyses provide a complementary perspective, specifically one that emphasizes continuity and hierarchy rather than modularity and segregation, offering insights into the integrative principles that underpin cognition and its disruption in disease.

Future research is poised to move in several exciting directions. First, moving beyond static, time-averaged gradients, a key challenge is to characterize their temporal dynamics and understand how they flexibly reconfigure on a moment-to-moment basis in response to cognitive demands. Second, establishing causality will require moving from correlational studies to causal interventions. Techniques such as non-invasive brain stimulation (e.g., TMS) targeted along gradient axes could directly test the causal role of this organization in behavior. A third major direction is to move beyond the cortex to develop a more integrated cortico-subcortical model. Structures like the thalamus, which acts as a central relay for cortical information flow, and the basal ganglia, critical for action selection, are intricately connected with the cortical mantle. Understanding how these subcortical nuclei align with and modulate cortical gradients will be essential for a complete model of brain function. Finally, the clinical translation of gradient-based metrics as biomarkers for diagnosis, prognosis, or treatment stratification remains a critical goal. Integrating this framework into clinical trials could provide mechanistic insights into why interventions succeed or fail. As neuroimaging methods advance and multimodal datasets expand, these gradient-based approaches will be essential for building a truly holistic model of brain function and dysfunction.
